# Supplementary material for: Mi-2β promotes immune evasion in melanoma by activating EZH2 methylation
Source: Nat Commun. 2024 Mar 9;15:2163. doi: 10.1038/s41467-024-46422-5 (PMC10924921; doi:10.1038/s41467-024-46422-5)
Supplement: Supplementary file 1 — Supplementary Information [file 41467_2024_46422_MOESM1_ESM.pdf]

Supplementary Information

Mi-2β promotes immune evasion in melanoma by activating EZH2 methylation

Cang Li[1,2#], Zhengyu Wang[3#], Licheng Yao[4#], Xingyu Lin[5], Yongping Jian[6], Yujia Li[6], Jie Zhang[7], Jingwei Shao[8], Phuc D. Tran[3], James R. Hagman[9], Meng Cao[10], Yusheng Cong[11], Hong-yu Li[3*], Colin R. Goding[12*], Zhi-Xiang Xu[6*], Xuebin Liao[4*], Xiao Miao[13*], Rutao Cui[1*]

Supplementary Table 1. Hazard ratio of epigenetic factor in melanoma patients depending on level of CD8 T infiltration

Gene	Hazard ratio (CD8 High)	P value (CD8 High)	Hazard ratio (CD8 Low)	P value(CD8 Low)
EP400	3.07773	0.00931	1.39484	0.19255
Mi-2β	3.04173	0.00506	0.94102	0.83052
PRDM4	2.96112	0.00881	1.21098	0.41654
USP7	2.65193	0.02903	0.997	0.99277
WDR5	2.43453	0.00579	1.49438	0.08988
EIF4A1	2.40095	0.02263	1.13934	0.57291
SMARCD1	2.378	0.00943	1.29621	0.26898
NCOA6	2.32242	0.01494	1.238	0.44929
CARM1	2.23837	0.034	1.50205	0.1032
SSRP1	2.13544	0.02357	0.83393	0.38984
NOP2	2.08097	0.00618	1.30647	0.16778
RCOR1	1.9907	0.0458	0.84654	0.46292
HDGF	1.95553	0.0193	1.15463	0.49071
RSAD1	1.92166	0.03828	1.08335	0.72936
FBL	1.91939	0.01392	1.15973	0.40459
HDAC4	1.834	0.01654	1.01963	0.91846
PARP1	1.80367	0.04806	1.47847	0.06788
RPS6KA4	1.74208	0.04125	1.34742	0.06752
IGHMBP2	1.51194	0.02636	1.06919	0.65526
CHD7	1.5025	0.02976	1.01916	0.90102
SKA1	1.48398	0.03118	1.12437	0.3458
TBL1X	1.4322	0.02111	0.93836	0.46205
HMGA1	1.39969	0.02909	0.99127	0.93907
SP140	0.76873	0.02862	0.87105	0.11047
ATM	0.7326	0.04831	0.78839	0.05981
HIST4H4	0.73247	0.02469	1.21279	0.06389
SMARCA1	0.73115	0.02881	1.00428	0.96361
SAP30	0.69911	0.0441	0.79723	0.07071
CENPQ	0.69442	0.0305	0.81165	0.07086
BAZ2B	0.68272	0.02704	0.83473	0.12737
PARP10	0.67194	0.009	0.97091	0.81199

CBX7	0.66918	0.00903	0.83659	0.0826
ZCWPW1	0.66456	0.01047	0.90748	0.33902
SP140L	0.64206	0.00364	0.88432	0.11526
NBN	0.62962	0.02439	0.79498	0.13523
DDX60L	0.62943	0.00117	0.83114	0.05277
MTF2	0.62824	0.01959	0.81613	0.16363
KAT2B	0.6195	0.00086	0.9079	0.30843
DTX3L	0.60908	0.00668	0.81064	0.08302
PARP14	0.57918	0.00246	0.7989	0.06022
DHX58	0.57911	0.00081	1.11798	0.30434
DZIP3	0.55709	0.0014	0.83752	0.21123
SP110	0.54961	0.00616	0.9442	0.70924
ZMYM6	0.5485	0.0119	0.7247	0.05437
AEBP2	0.52732	0.00836	0.85752	0.42023
BRD7	0.51796	0.0237	0.81917	0.29759
ING4	0.51618	0.0281	0.74547	0.13177
PARP9	0.51025	0.00019	0.85879	0.16677
SP100	0.50655	0.00097	0.84107	0.19737
PHF1	0.49778	0.01862	0.95018	0.78662
H2AFV	0.48423	0.03085	1.1064	0.67233
CBX3	0.47037	0.00144	0.91017	0.61197
DPY30	0.43034	0.03116	0.81318	0.39719
PHF12	0.41747	0.04267	0.92898	0.78398
HP1BP3	0.41218	0.01638	1.10296	0.72258
EP400	3.07773	0.00931	1.39484	0.19255

hazard ratio derived from Cox regression test, Statistical analysis was conducted using log-rank tests.

Supplementary Table 2. Top 10 GSEA-Hallmark upregulated genes

Gene Set Name	# Genes in Gene Set (K)	# Genes in Overlap (k)	k/K	p-value	FDR q-value
HALLMARK_INTERFERON_GAMMA_RESPONSE	200	66	0.33	4.15E-47	2.08E-45
HALLMARK_TNFA_SIGNALING_VIA_NFKB	200	63	0.315	1.31E-43	3.28E-42
HALLMARK_INTERFERON_ALPHA_RESPONSE	97	41	0.423	7.5E-35	1.25E-33
HALLMARK_HYPOXIA	200	46	0.23	1.29E-25	1.61E-24
HALLMARK_INFLAMMATORY_RESPONSE	200	38	0.19	2.35E-18	2.35E-17
HALLMARK_MTORC1_SIGNALING	200	37	0.185	1.65E-17	1.37E-16
HALLMARK_XENOBIOTIC_METABOLISM	200	35	0.175	7.35E-16	5.25E-15
HALLMARK_EPITHELIAL_MESENCHYMAL_TRANSITION	200	33	0.165	2.85E-14	1.78E-13
HALLMARK_HEME_METABOLISM	200	32	0.16	1.68E-13	9.35E-13
HALLMARK_IL2_STAT5_SIGNALING	200	31	0.155	9.57E-13	4.79E-12

P value is the gene set enrichment test result; FDR q value is the p-value corrected by multiple hypothesis testing.

Supplementary Table 3. Top 10 GSEA-Hallmark downregulated genes

Gene Set Name	# Genes in Gene Set (K)	# Genes in Overlap (k)	k/K	p-value	FDR q-value
HALLMARK_G2M_CHECKPOINT	200	50	0.25	9.96E-30	4.98E-28
HALLMARK_E2F_TARGETS	200	48	0.24	9.97E-28	2.49E-26
HALLMARK_MITOTIC_SPINDLE	199	47	0.236	7.55E-27	1.26E-25
HALLMARK_EPITHELIAL_MESENCHYMAL_TRANSITION	200	46	0.23	8.92E-26	1.12E-24
HALLMARK_GLYCOLYSIS	200	37	0.185	1.24E-17	1.24E-16
HALLMARK_APICAL_JUNCTION	200	31	0.155	7.63E-13	5.45E-12
HALLMARK_KRAS_SIGNALING_UP	200	31	0.155	7.63E-13	5.45E-12

HALLMARK_ESTROGEN_RESPONSE_LATE	200	29	0.145	2.24E-11	1.4E-10
HALLMARK_COAGULATION	138	21	0.152	5.19E-09	2.88E-08
HALLMARK_MYOGENESIS	200	25	0.125	1.19E-08	5.94E-08

P value is the gene set enrichment test result; FDR q value is the p-value corrected by multiple hypothesis testing.

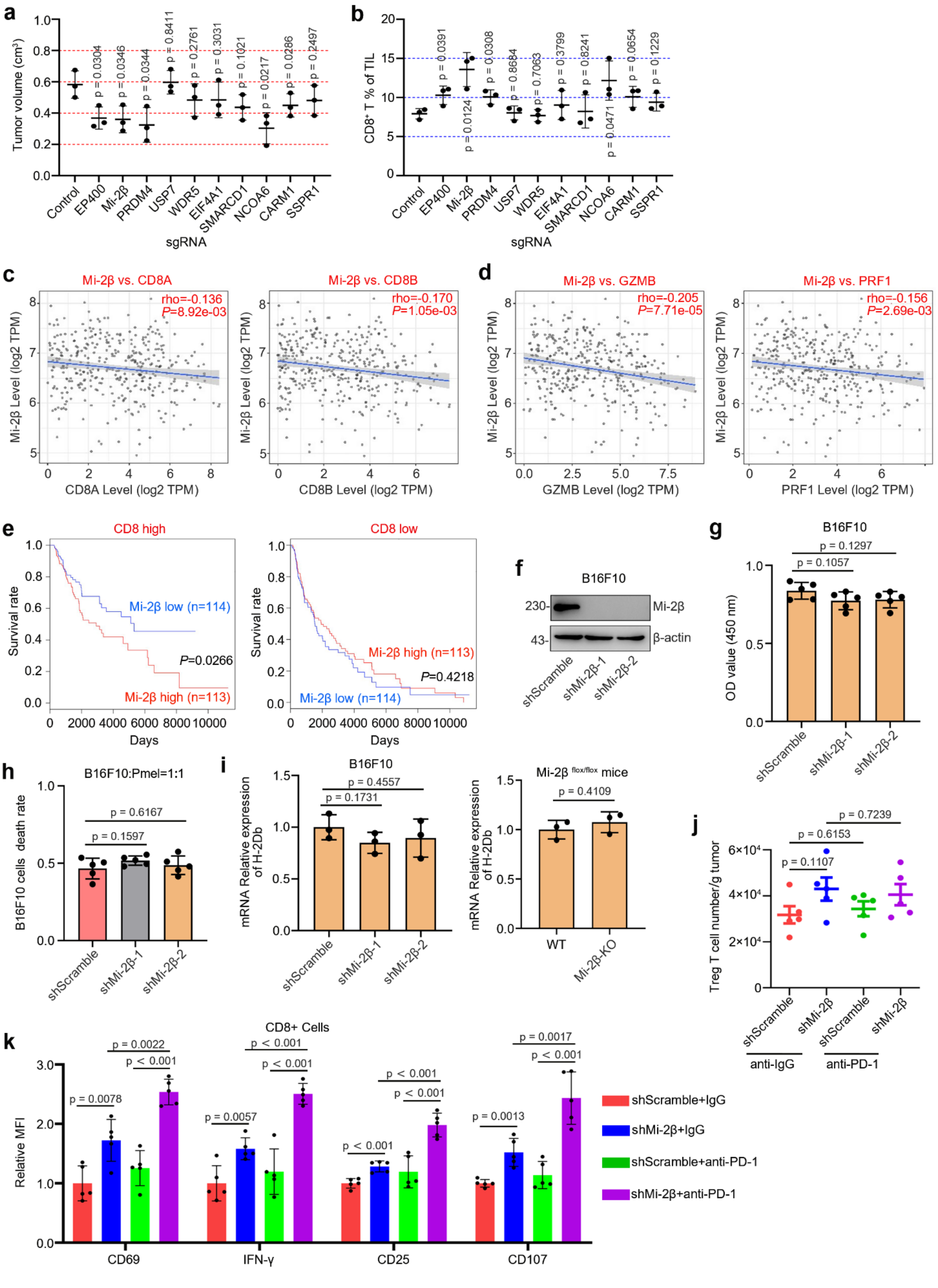
a
b
c
d
e
f
g
h
i
j
k
Tumor volume (cm³)
CD8⁺ T % of TIL
sgRNA
Mi-2β vs. CD8A
Mi-2β vs. CD8B
Mi-2β vs. GZMB
Mi-2β vs. PRF1
CD8 high
CD8 low
Mi-2β low (n=114)
Mi-2β high (n=113)
P=0.0266
P=0.4218
B16F10
Mi-2β
β-actin
shScramble
shMi-2β-1
shMi-2β-2
OD value (450 nm)
B16F10:Pmel=1:1
B16F10 cells death rate
mRNA Relative expression of H-2Db
Mi-2β flox/flox mice
WT
Mi-2β-KO
Treg T cell number/g tumor
anti-IgG
anti-PD-1
CD8+ Cells
Relative MFI
CD69
IFN-γ
CD25
CD107
shScramble+IgG
shMi-2β+IgG
shScramble+anti-PD-1
shMi-2β+anti-PD-1

Supplementary Figure 1. Hazard ratio of epigenetic factors dependent on CD8 T cell infiltration in melanoma. a Tumor volume was measured after each candidate gene was silenced by specific gRNA in B16F10 cells and grafted into C57BL6 mice (n=3). **b** TILs were measured by flow cytometry. The relative numbers of $CD8^+$ gated within $CD45^+$ cells were shown. **c** Analysis of correlation between Mi-2β mRNA level and CD8A or CD8B as T cell infiltration markers in TCGA SKCM-Metastasis (n=368). Plots show the Spearman's correlation. **d** Analysis of correlation between Mi-2β mRNA level and GZMB or PRF1 level as cytotoxicity markers. Analysis was performed as indicated. **e** The survival curve of melanoma patients with different Mi-2β mRNA levels. All patients in TCGA melanoma were divided into CD8 high or CD8 low groups based on the median expression. The available patients were further split into high- or low-expressing groups according to the median of Mi-2β mRNA level expression. Kaplan-Meier survival curves were shown, with the difference was examined using a log-rank test. **f** Western blot assay showing the efficiency of shMi-2β knockdown in B16F10 cells (n = 3, independent experiments). **g** Detection of tumor cell proliferation in B16-F10 cells with Mi-2β silencing by CCK-8 (n=5). **h** Detection of the sensitivity to Pmel-1 T killing after Mi-2β silencing (n=5). **i** The expression of H-2Db was measured in B16F10 cells and mice tumor cells after Mi-2β silencing by RT-qPCR assay (n=3). **j** The number of Treg cells within CD45+ T cells was demonstrated (n=5). **k** Expression of activation markers of $CD8^+$ T cells were measured by flow cytometry (n=5). MFI represents mean fluorescence intensity. **a, b, j, h, i, j, k:** Values represent mean ± SD. The unpaired, two tailed t-test. Source data are provided as a Source Data file.

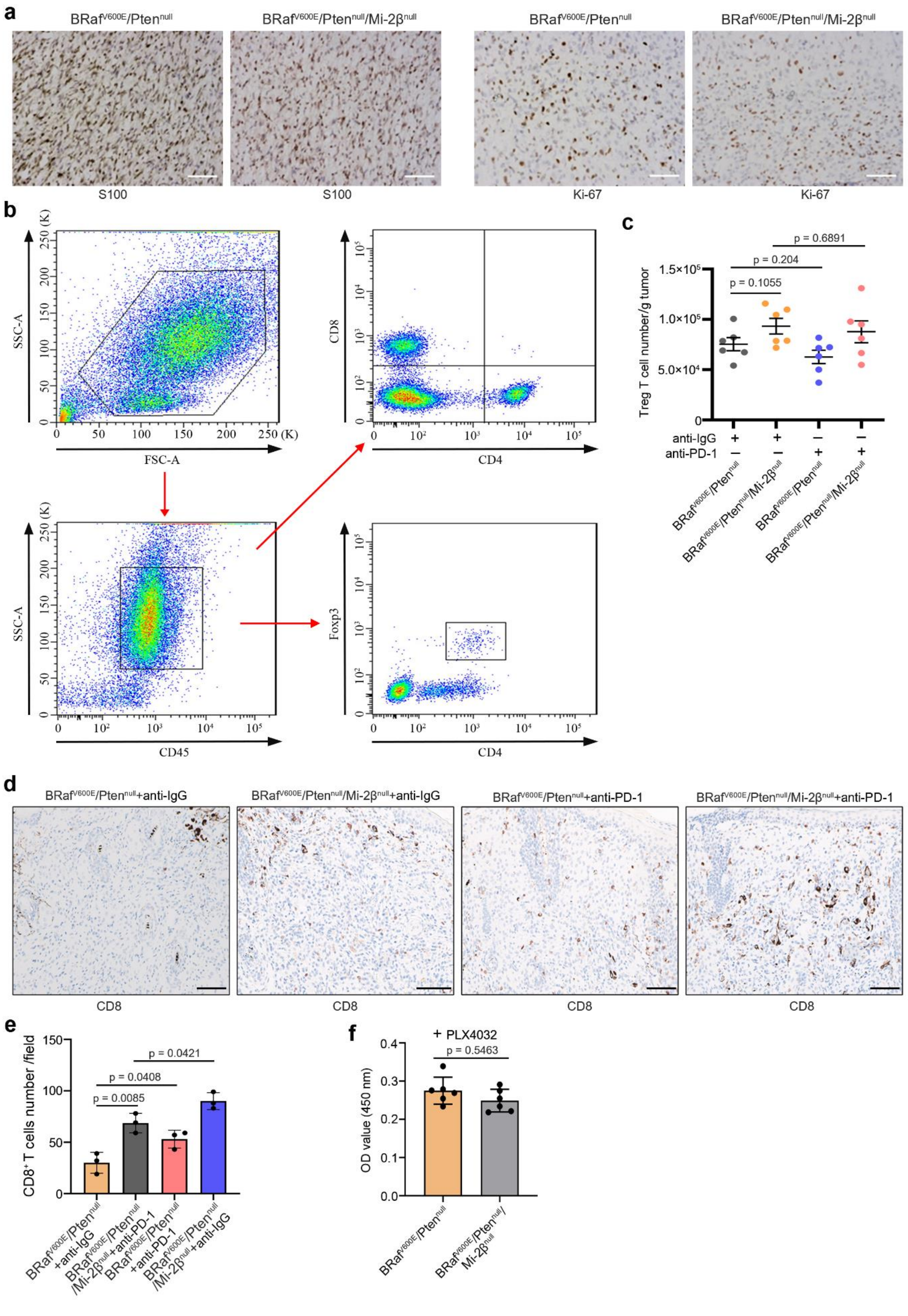
a
BRafV600E/Ptennull
BRafV600E/Ptennull/Mi-2βnull
S100
Ki-67
b
SSC-A
FSC-A
CD8
CD4
CD45
Foxp3
c
p = 0.6891
p = 0.204
p = 0.1055
Treg T cell number/g tumor
anti-IgG
anti-PD-1
d
BRafV600E/Ptennull+anti-IgG
BRafV600E/Ptennull/Mi-2βnull+anti-IgG
BRafV600E/Ptennull+anti-PD-1
BRafV600E/Ptennull/Mi-2βnull+anti-PD-1
CD8
e
CD8+ T cells number /field
p = 0.0421
p = 0.0408
p = 0.0085
f
+ PLX4032
p = 0.5463
OD value (450 nm)

Supplementary Figure 2. Analysis of Mi-2β deficient melanoma. a Melanomas collected from $BRaf^{V600E}/Pten^{null}$ mice and $BRaf^{V600E}/Pten^{null}$ /Mi-$2\beta^{null}$ mice were prepared and processed for immunohistochemistry staining to detect the expression of the melanoma marker S100 and tumor proliferation marker Ki-67. Scale bar=200 µm. **b** Tumor-infiltrating lymphocytes were assayed by flow cytometry. **c** Treg cells within $CD45^{+}$ T cells in TILs were assayed and quantified by flow cytometry. (n=5). **d** Melanomas isolated from $BRaf^{V600E}/Pten^{null}$ mice and $BRaf^{V600E}/Pten^{null}$ /Mi-$2\beta^{null}$ mice were processed for immunohistochemistry staining to detect the number of CD8+ T cells. Scale bar=200 µm. **e** Absolute number of CD8+ T cells in per field (n=3). **f** Detection of tumor cell proliferation after PLX4032 treatment. Melanoma cells isolated from *Tyr::CreER;BRaf*^{CA}*;Pten*$^{lox/lox}$ and *Tyr::CreER;BRaf*^{CA}*;Pten*$^{lox/lox}$*;Mi-2β*$^{lox/lox}$ mice were treated with PLX4032 (10µM) for 24 hours and cell proliferation was determined by CCK-8 assay (n=6). **c, e, f:** Values represent mean ± SD. The unpaired, two tailed t-test. Source data are provided as a Source Data file.

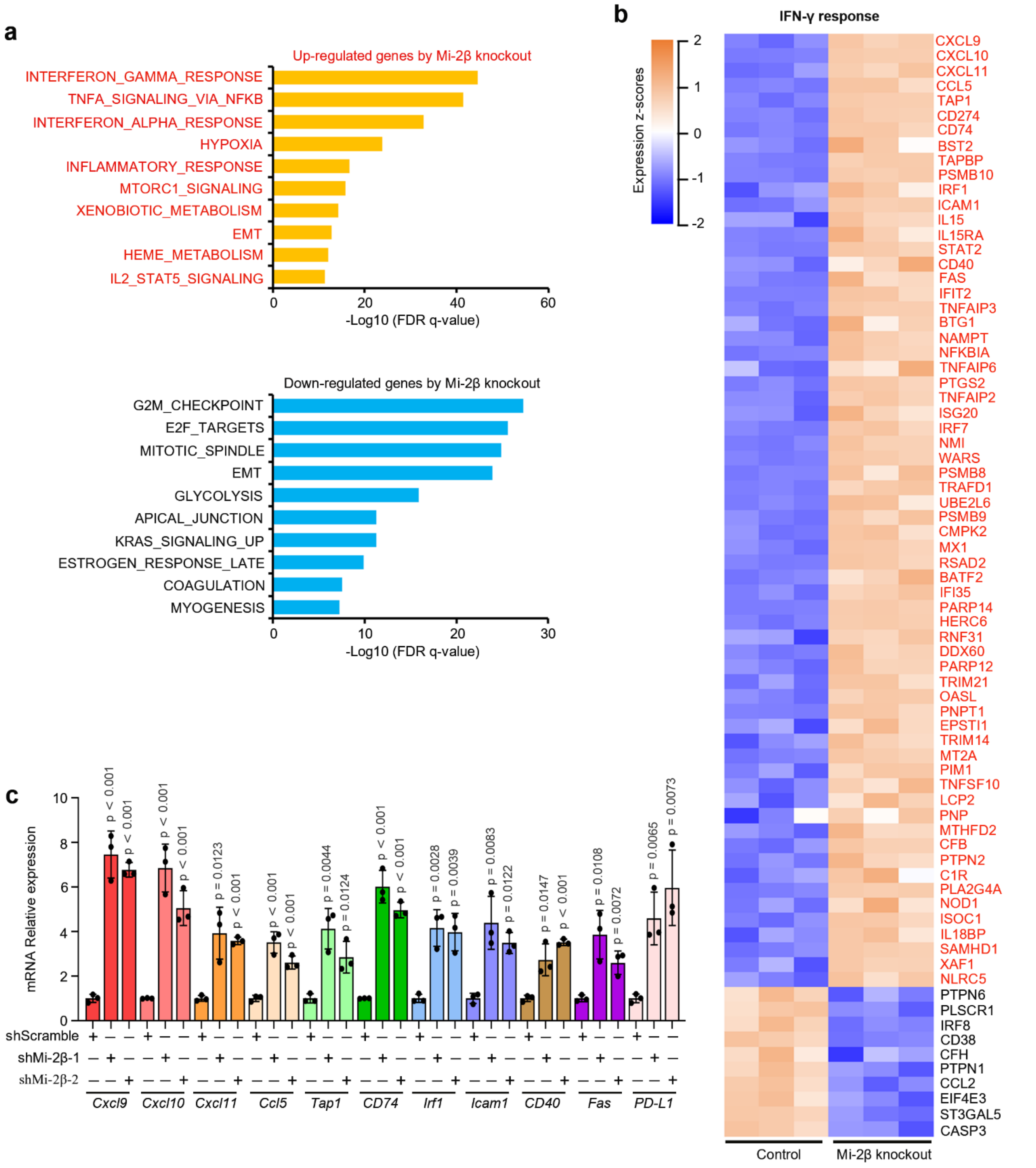

Supplementary Figure 3. Enhanced IFN-γ signaling in Mi-2β knockout melanomas. a Microarray data analyzed for hallmark gene sets enriched for upregulated or downregulated mRNA in Mi-2β knockout and control B16F10 cells treated with IFN-γ for 24 hours. **b** Heat map showing expression value (z-score expression) of IFN-γ signaling genes in control and Mi-2β knockout B16F10 cells in microarray data. **c**

The expression of Mi-2β-regulated IFN-γ signaling genes were measured in IFN-γ-stimulated B16F10 cells with Mi-2β silencing by RT-qPCR assay (n=3). Values represent mean ± SD. The unpaired, two tailed t-test. Source data are provided as a Source Data file.

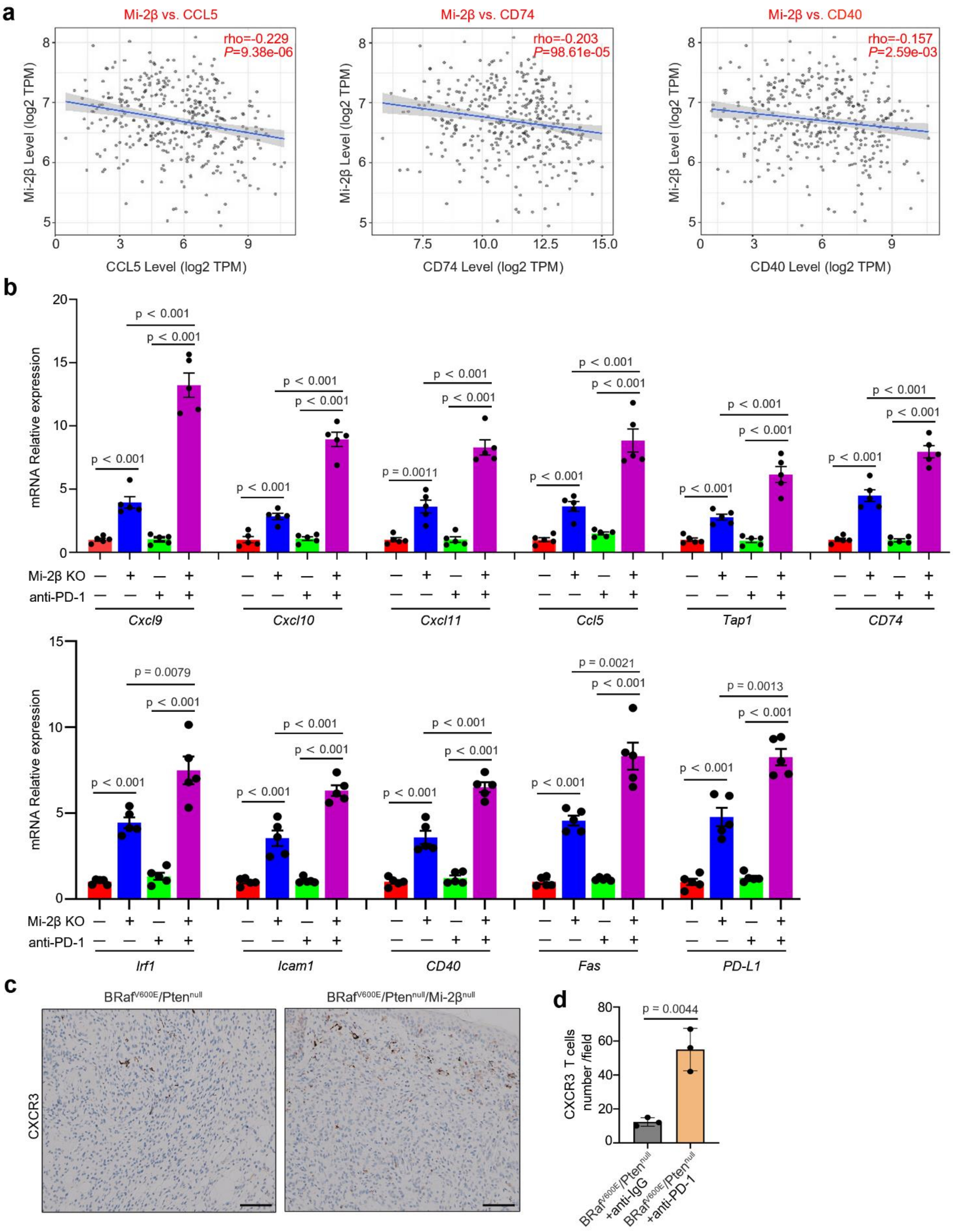
a
Mi-2β vs. CCL5
rho=-0.229
P=9.38e-06
Mi-2β vs. CD74
rho=-0.203
P=98.61e-05
Mi-2β vs. CD40
rho=-0.157
P=2.59e-03
Mi-2β Level (log2 TPM)
CCL5 Level (log2 TPM)
CD74 Level (log2 TPM)
CD40 Level (log2 TPM)
b
mRNA Relative expression
Mi-2β KO
anti-PD-1
Cxcl9
Cxcl10
Cxcl11
Ccl5
Tap1
CD74
Irf1
Icam1
CD40
Fas
PD-L1
p < 0.001
p = 0.0011
p = 0.0079
p = 0.0021
p = 0.0013
c
BRaf V600E/Pten null
BRaf V600E/Pten null/Mi-2β null
CXCR3
d
CXCR3 T cells number /field
p = 0.0044
BRaf V600E/Pten null +anti-IgG
BRaf V600E/Pten null +anti-PD-1

Supplementary Figure 4. Mi-2β directly regulates inflammatory genes. a Plots showed the Spearman's correlation between Mi-2β mRNA level and CCL5, CD74 or CD40 mRNA expression level in RNA-seq data in TCGA SKCM-Metastasis (n=368). **b** The Mi-2β-regulated downstream target genes implicated in IFN-γ signaling were measured in $BRaf^{V600E}/Pten^{null}$ and $BRaf^{V600E}/Pten^{null}$ /Mi-$2\beta^{null}$ melanoma in mice treated with IgG control or anti-PD-1 with RT-qPCR assay (n=5). Values represent mean ± SEM. **c** Melanomas collected from $BRaf^{V600E}/Pten^{null}$ mice and $BRaf^{V600E}/Pten^{null}$ /Mi-$2\beta^{null}$ mice were fixd and processed for immunohistochemistry staining to detect the expression of CXCR3. Scale bar = 200 µm. **d** Absolute number of CXCR3 T cells in per field (n=3). Values represent mean ± SD. **b, d:** The unpaired, two tailed t-test. Source data are provided as a Source Data file.

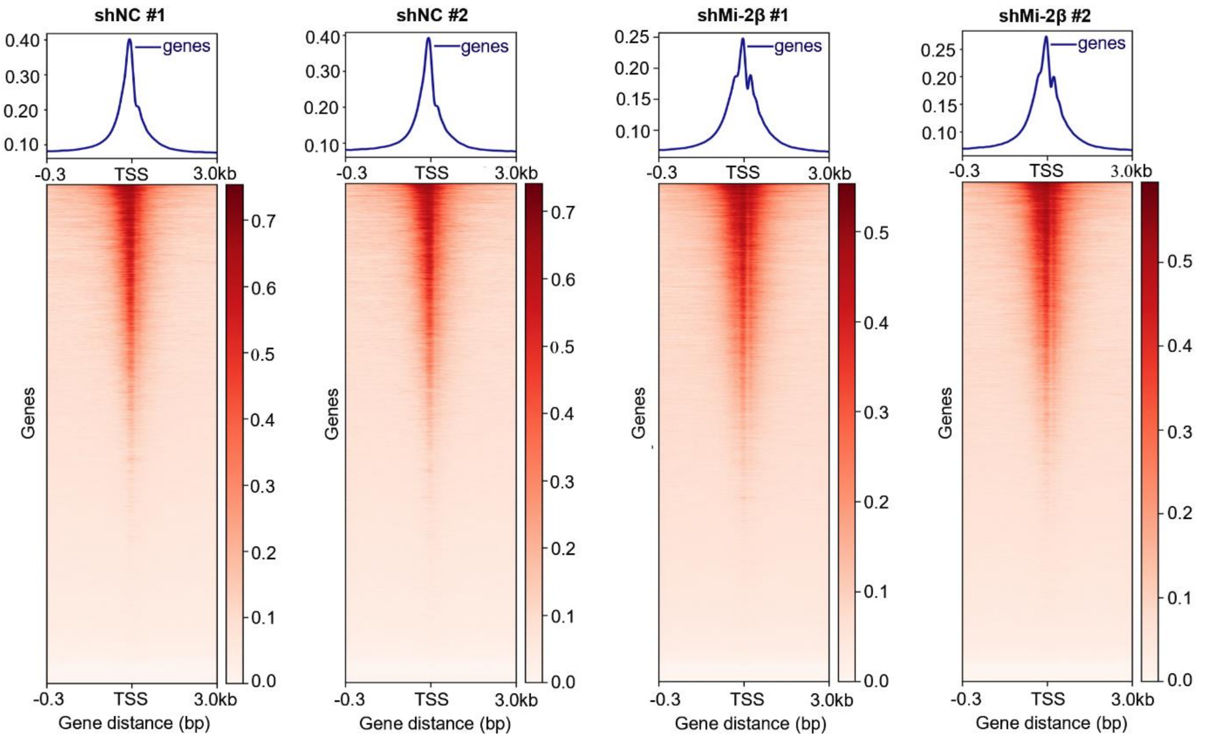

Supplementary Figure 5. Depletion of Mi-2β promotes the transcription of interferon stimulated genes by promoting chromatin remodeling. Average ATAC-seq signal distribution near the TSS. Source data are are available in the GEO database under accession code is GSE255782..

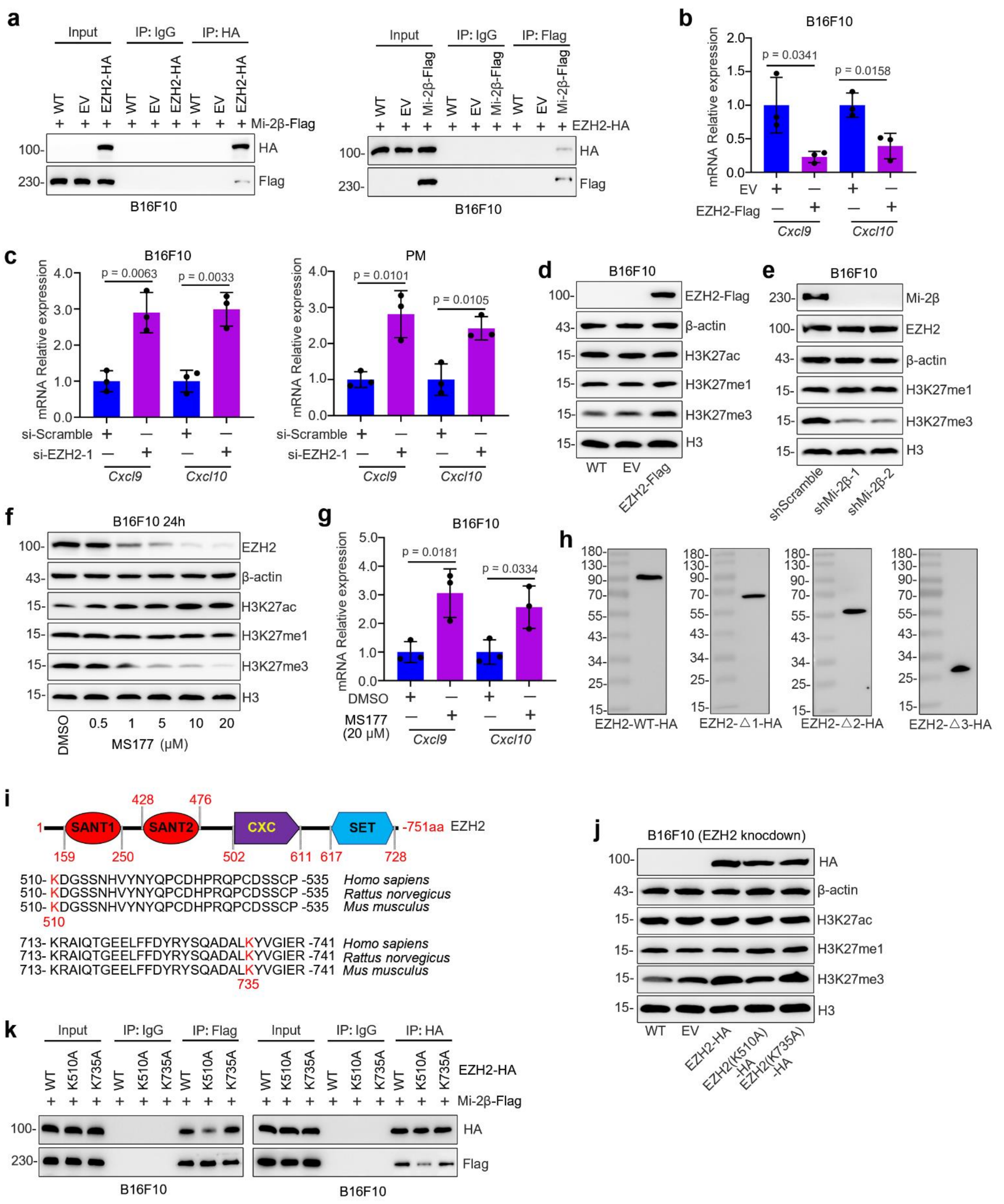

Supplementary Figure 6. EZH2 silencing represses H3K27me3 and activates interferon-stimulated gene expression. a Exogenous interactions between Mi-2β-Flag and EZH2-HA were detected by

immunoprecipitation in B16F10 cells (n = 3, independent experiments). **b** The mRNA expression of Mi-2β-regulated IFN-γ signaling genes (*Cxcl9* and *Cxcl10*) was measured by qRT-PCR in B16F10 cells after EZH2 overexpression (n=3). **c** The mRNA expression of Mi-2β-regulated IFN-γ signaling genes (*Cxcl9* and *Cxcl10*) was measured by qRT-PCR in B16F10 or PM (Primary mouse melanoma) cells with EZH2 silencing (n=3). **d** The epigenetic modification of H3 (H3K27ac, H3K27me1 and H3K27me3) were measured by Western blot in B16F10 cells with EZH2 overexpression (n = 3, independent experiments). **e** The epigenetic modifications of H3 (H3K27me1 and H3K27me3) were measured in B16F10 cells with Mi-2β silencing by Western blot (n = 3, independent experiments). **f** The epigenetic modifications of H3 (H3K27ac, H3K27me1 and H3K27me3) were measured in B16F10 cells or PM cells after stimulated with EZH2 inhibitor MS177 (n = 3, independent experiments). **g** The mRNA expressions of Mi-2β-regulated IFN-γ signaling genes were measured by qRT-PCR in B16F10 cells or PM cell stimulated with EZH2 inhibitor MS177 (n=3). **h** HA-tagged EZH2 WT or deletion mutant protein were measured in B16F10 cells by Western blot (n = 2, independent experiments). **i** A schematic of domains of EZH2 from different species. Methylation sites at K510 and K735 are highlighted. **j** The epigenetic modifications of H3 (H3K27ac, H3K27me1 and H3K27me3) were measured in B16F10 cells with stable EZH2 silencing and EZH2 or mutation EZH2 reintroduction by Western blot (n = 3, independent experiments). **k** Exogenous interactions between Mi-2β and EZH2 or mutated EZH2 were detected by immunoprecipitation in B16F10 cells (n = 3, independent experiments). **b, c, g:** Values represent mean ± SD. The unpaired, two tailed t-test. Source data are provided as a Source Data file.

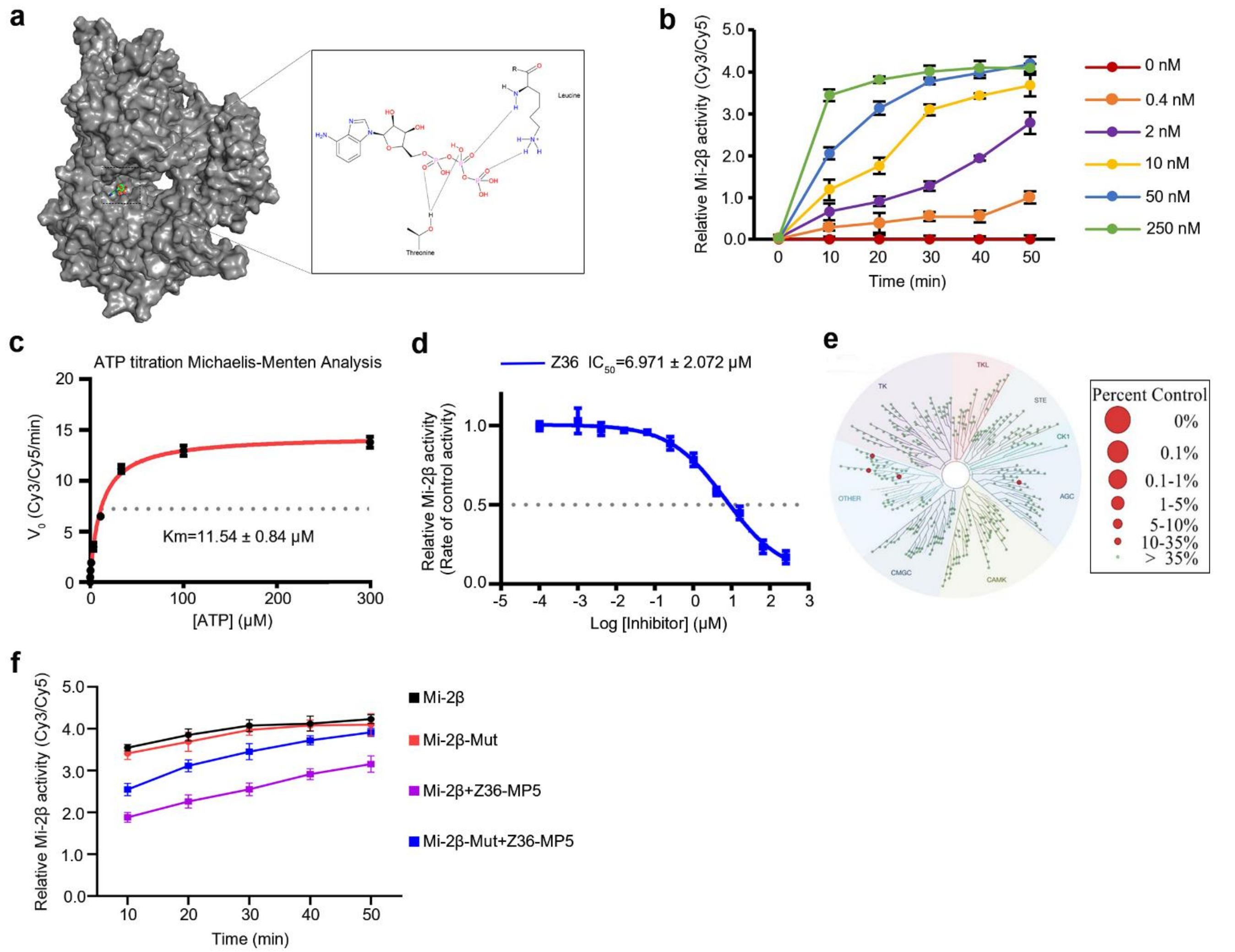

Supplementary Figure 7. *In vitro* nucleosome remodeling reactions. a The candidate protein structure for homology modelling. 3MWY depicted the interaction of ATP and its binding pocket. **b** The FRET-based nucleosome repositioning assays were performed with different concentrations of Mi-2β and a non-limiting ATP concentration (1 mM) for the indicated incubation time (n=3). **c** The ATP titration (concentrations ranging from 0.1 to 300 μM) was performed with the FRET-based nucleosome repositioning assays. The Michaelis-Menten equation was performed to calculate the apparent ATP Km, with the ATP Km of 11.54 μM (n=3). Values represent mean ± SD. **d** The inhibitory activity of Z36 for Mi-2β chromatin modulatory activity, measured as fold change of Mi-2β activity compared to those treated with control vehicle (n=3). **e** KINOMEscan profiles of compound Z36-MP5. KINOMEscan profiling of Z36-MP5 at a concentration of 1000 nM against 468 kinases. **f** The FRET-based nucleosome repositioning assays were performed with Mi-2β or Mi-2β mutation (H727A) for the indicated incubation time. ATP concentration is 1 mM and the Z36-MP5 concentration is 10 μM (n=3). Values represent mean ± SD. Source data are provided as a Source Data file.

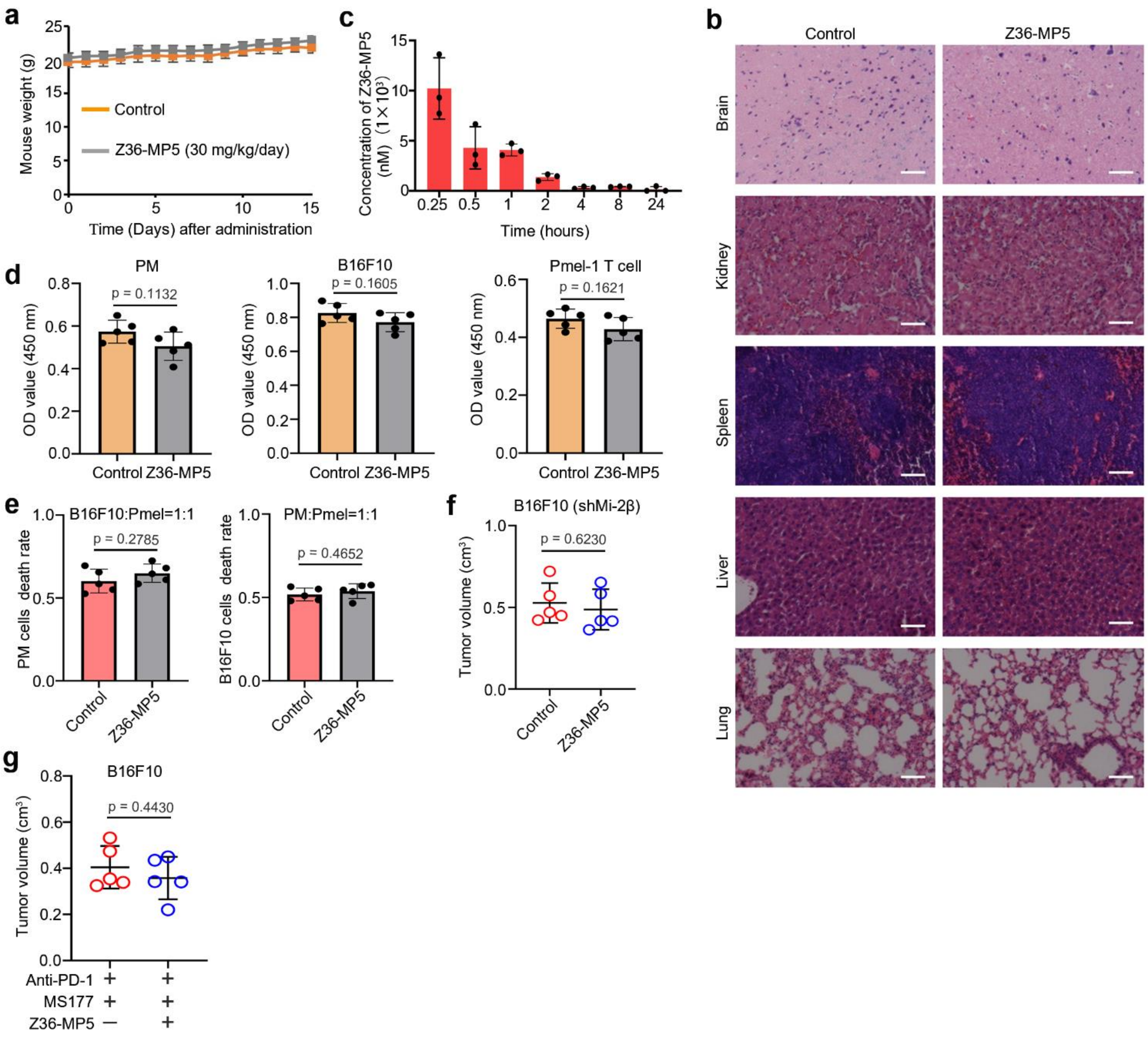

Supplementary Figure 8. ***In vitro*** **assay for Mi-2β inhibitors.** **a** The body weight changes of C57BL/6J mice treated with Z36-MP5 (30 mg/kg/day) for 2 weeks. Data are mean ± SEM (n=5). **b** H&E staining of tissues in C57BL/6J mice treated with or without Z36-MP5 (30 mg/kg/day) for 2 weeks. Scale bar=200 μm. **c** Blood concentration profiles of Z36-MP5 after a single-dose intraperitoneal injection into 3 male Sprague-Dawley (SD) rats. **d** Detection of cell proliferation in B16-F10 cells treated with Z36-MP5 (50 μM) using CCK-8 (n=5). **e** Detection of the sensitivity to Pmel-1 T killing after Z36-MP5 (50 μM) treatment (n=5). **f** Mice bearing B16F10 cells (shMi-2β) were treated with Z36-MP5 (30 mg/kg/day) or vehicle, accompanied with anti-PD-1 antibody (10 mg/kg, 3, 6, 9, 12, 15 days after bearing B16F10 cells). The growth of tumor grafts was measured. n = 5. **g** Mice bearing B16F10 cells were treated with MS177 (100 mg/kg, 5 days) and then Z36-MP5 (30 mg/kg/day) or vehicles, accompanied with anti-PD-1 antibody

(10 mg/kg/3 days). The growth of tumor grafts was measured. n = 5. **d, e, f, g:** Values represent mean ± SD. The unpaired, two tailed t-test. Source data are provided as a Source Data file.

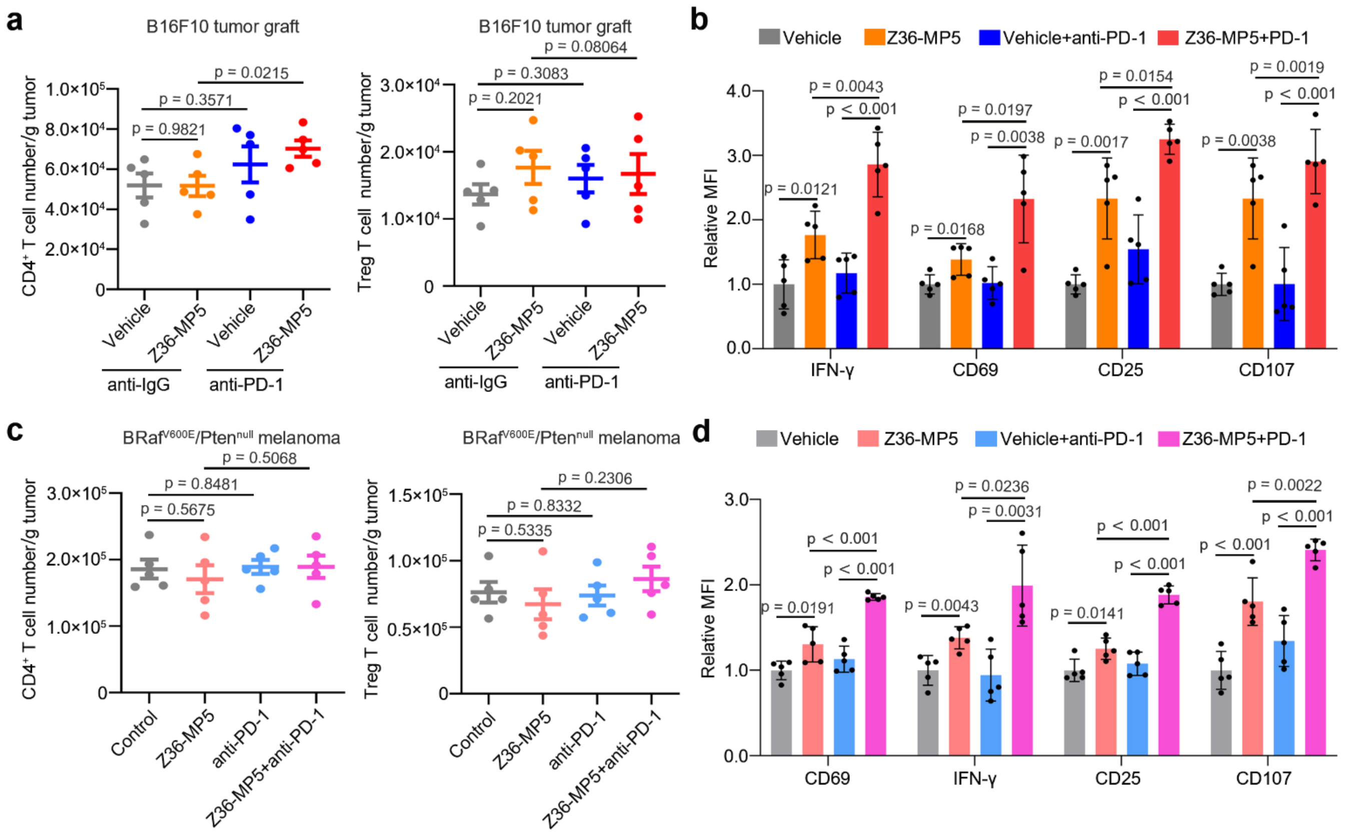

Supplementary Figure 9. Combinational treatment of Z36-MP5 and anti-PD-1. a Tumor-infiltrating lymphocytes (TILs) were assayed and quantified for measuring the number of $CD4^+$ and Treg cell populations in $CD45^+$ cells with flow cytometry (n = 5). **b** Expression of activation markers on $CD8^+$ T cells were determined and quantified with flow cytometry assay (n = 5). **c** Transgenic mice expressing $BRaf^{V600E}/Pten^{null}$ or $BRaf^{V600E}/Pten^{null}$ /Mi-$2\beta^{null}$ with measurable tumors were randomly treated with either control IgG (10 mg/kg) or anti-PD-1 antibody (10 mg/kg) and Z36-MP5 (30 mg/kg/day) or vehicle control by i.p. administration. For each group n = 5. TILs were assayed by flow cytometry. The number of tumor-infiltrating $CD4^+$ T cells and Treg cells gated within $CD45^+$ T cells were assayed and quantified with flow cytometry. **d** The activation markers on $CD8^+$ T cells were determined and quantified with flow cytometry assay. MFI, mean fluorescence intensity. (n = 5). **a, b, c, d:** Values represent mean ± SD. The unpaired, two tailed t-test. Source data are provided as a Source Data file.

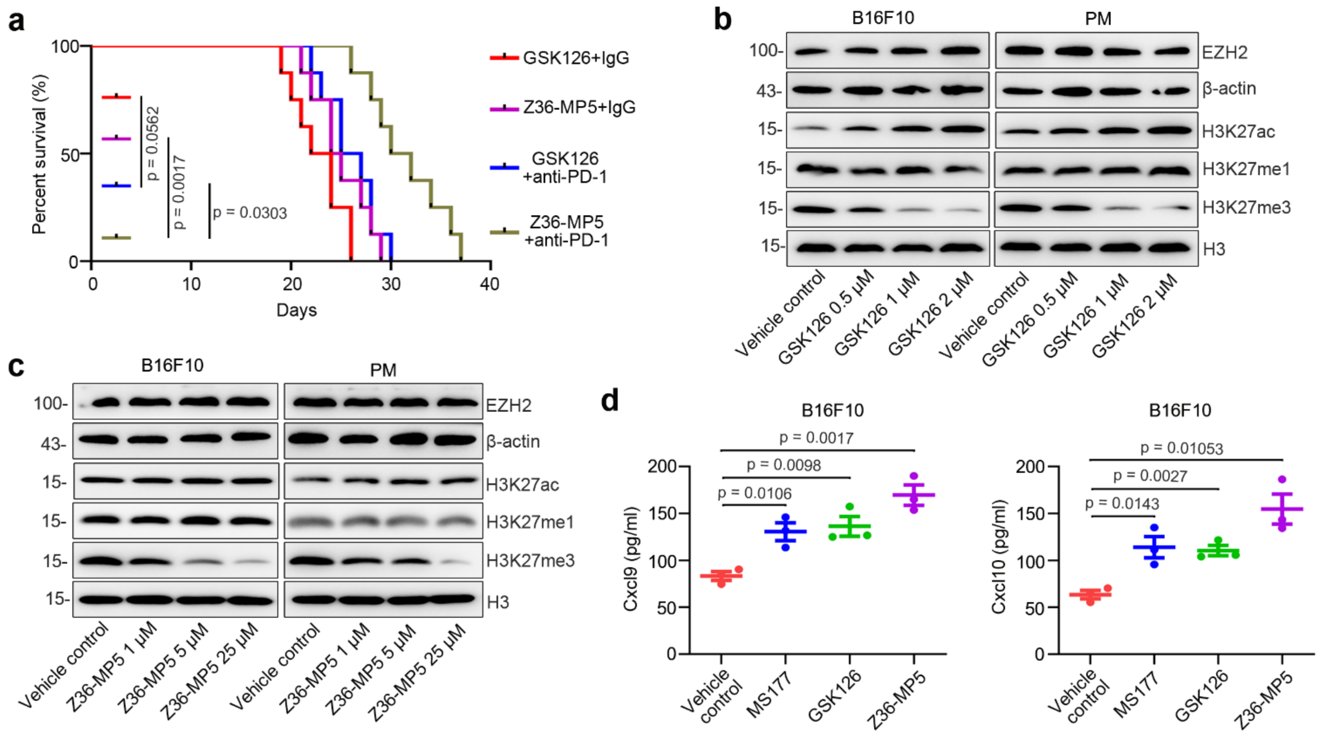

Supplementary Figure 10. Comparison of tumor growth inhibition effects between Z36-MP5 and EZH2 inhibitors. a Mice (n=8) bearing B16F10 cells were treated with control IgG or anti-PD-1 antibody in combination with Z36-MP5 or GSK126, as indicated. A mouse survival curve is shown, with log-rank test for mouse survival *P* value. **b,c** The epigenetic modifications of H3 (H3K27ac, H3K27me1 and H3K27me3) were measured in B16F10 cells stimulated with GSK126 or Z36-MP5 as indicated (n = 3, independent experiments). **d** The amount of secreted Cxcl9 and Cxcl10 was measured by ELISA in B16F10 cells stimulated with MS177 (10 μM), GSK126 (2μM) or Z36-MP5 (10 μM) (n=3). Values represent mean ± SD. The unpaired, two tailed t-test. Source data are provided as a Source Data file.

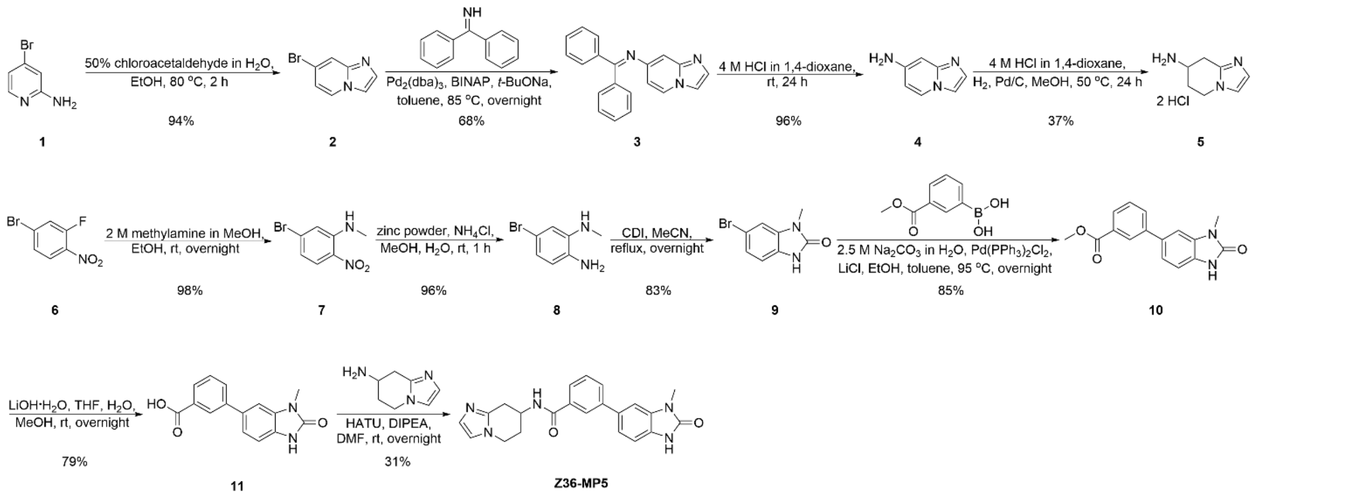

Supplementary Figure 11. Chemical synthesis Z36-MP5.

(a) 50% chloroacetaldehyde in H_2O, EtOH, 80 °C, 2 h; (b) benzophenone imine, $Pd_2(dba)_3$, BINAP, *t*-BuONa, toluene, 85 °C, overnight; (c) 4 M HCl in 1,4-dioxane, room temperature, 24 h; (d) 4 M HCl in 1,4-dioxane, Pd/C, MeOH, 50 °C, 24 h; (e) 2 M methylamine solution in MeOH, EtOH, room temperature, overnight; (f) zinc powder, NH_4Cl, H_2O, MeOH, room temperature, 1 h; (g) carbonyldiimidazole, ACN, reflux, overnight; (h) 3-methoxycarbonylphenylboronic acid, 2.5 M Na_2CO_3, $Pd(PPh_3)_2Cl_2$, LiCl, EtOH, toluene, sealed tube, 95 °C, overnight; (i) LiOH·H_2O, THF, MeOH, H_2O, rt, overnight; (j) **5**, HATU, DIPEA, DMF, rt, overnight.

Step a: synthesis of 7-bromoimidazo[1,2-a]pyridine, ***2***

A mixture of 4-bromopyridin-2-amine (**1**, 10.4 g, 60.0 mmol) and 50% chloroacetaldehyde in H_2O (18.8 g, 120.0 mmol) in EtOH (150.0 mL) was stirred at 75 °C for 2 hours. After the complete conversion detected by TCL analysis (DCM : MeOH = 10 : 1), the reaction mixture was concentrated under vacuum to afford a yellow thick oil. EA (50.0 mL) was added to the thick oil and the resulting suspension was stirred at room temperature for 30 minutes to generate a yellow suspension. Then the suspension was filtered to afford a light yellow solid which was washed with EA (20.0 mL) and hexanes (20.0 mL) to afford an off-white solid as 7-bromoimidazo[1,2-*a*]pyridine (**2**, 11.1 g, 93.7% yield). LC-MS: 197.12 $[M]^+$.

Step b: synthesis of N-(imidazo[1,2-a]pyridin-7-yl)-1,1-diphenylmethanimine, ***3***

A mixture of 7-bromoimidazo[1,2-*a*]pyridine (**2**, 0.4 g, 2.0 mmol), benzophenone imine (0.7 g, 4.0 mmol), *t*-BuONa (0.4 g, 4.0 mmol), $Pd_2(dba)_3$ (92.0 mg, 0.1 mmol), and BINAP (93.0 mg, 0.15 mmol) in toluene (10.0 mL) was degassed with N_2 for 15 minutes. Then the reaction mixture was allowed to stir at 85 °C overnight. After cooling to room temperature, the resulting mixture was diluted with water (50.0 mL) and extracted with EA (50.0 mL * 3). The combined organic layers were washed with brine (50.0 mL), dried over anhydrous Na_2SO_4, filtered, concentrated under vacuum, absorbed onto silica gel, and purified via flash chromatography (DCM : MeOH = 30 : 1) to afford a yellow oil as *N*-(imidazo[1,2-*a*]pyridin-7-yl)-1,1-diphenylmethanimine (**3**, 0.4 g, 69% yield). LC-MS: 297.40 $[M]^+$.

Step c: synthesis of imidazo[1,2-a]pyridin-7-amine, ***4***

A solution of *N*-(imidazo[1,2-*a*]pyridin-7-yl)-1,1-diphenylmethanimine (**3**, 0.4 g, 1.4 mmol) in 4 M hydrogen chloride solution in 1,4-dioxane was stirred at room temperature for 24 hours to afford a dark

brown suspension. After the complete conversion detected by LC-MS analysis, the resulting mixture was filtered to obtain a brown solid which was washed with DCM (5.0 mL) to afford a dark yellow solid. The dark yellow solid was dissolved in MeOH (10.0 mL), aborbed onto celite, and purified via C18 reversed-phase flash column chromatography (H_2O : MeOH = 9 : 1) to afford a brown solid as imidazo[1,2-a]pyridin-7-amine (**4**, 0.15 g, 56% yields). LC-MS: 133.41 $[M]^+$.

*Step d: synthesis of 5,6,7,8-tetrahydroimidazo[1,2-a]pyridin-7-amine dihydrochloride, **5***

A mixture of imidazo[1,2-a]pyridin-7-amine (**4**, 0.1 g, 0.78 mmol), Pd/C (20.0 mg, 20%wt), and 4 M hydrogen chloride solution in 1,4-dioxane (0.2 mL) in MeOH (5.0 mL) was stirred at 50 ^oC for 24 hours under H_2 atmosphere. After the complete conversion detected by TLC (DCM: MeOH = 10 : 1) and LC-MS analysis, the resulting mixture was concentrated under vacuum to afford a yellow solid. DCM (5.0 mL) was added to the yellow solid and the resulting suspension was stirred at room temperature for 15 minutes to generate a light yellow suspension. Then the suspension was filtered to afford a light yellow solid which was washed with a combined solution of DCM and MeOH (DCM: MeOH = 10:1, 5 mL) to afford an beige solid as 5,6,7,8-tetrahydroimidazo[1,2-a]pyridin-7-amine dihydrochloride (**5**, 0.1 g, 61% yield). LC-MS: 137.10 $[M]^+$.

*Step e: synthesis of 5-bromo-N-methyl-2-nitroaniline, **7***

To a solution of 4-bromo-2-fluoro-1-nitrobenzene (**6**, 4.4 g, 20.0 mmol) in EtOH (50.0 mL) was added 2 M methylamine solution in MeOH (12.0 mL, 240.0 mmol). The reaction mixture was stirred at room temperature overnight. After the complete conversion detected LC-MS analysis, the resulting mixture was concentrated under vacuum, and the residual orange solid was partitioned between water (200.0 mL) and EA (200.0 mL * 3). The combined organic phases were washed with brine, dried over anhydrous Na_2SO_4, filtered, concentrated under vacuum to afford a bright orange solid as 5-bromo-*N*-methyl-2-nitroaniline (**7**, 4.4 g, 97% yield). LC-MS: 232.30 $[M + H]^+$.

*Step f: synthesis of 5-bromo-N^1-methylbenzene-1,2-diamine, **8***

To a suspension of 5-bromo-*N*-methyl-2-nitroaniline (**7**, 3.0 g, 13.0 mmol) and ammonium chloride (7.0 g, 130.0 mmol) in MeOH (17.0 mL) and water (35.0 mL) was added zinc powder (4.2 g, 65.0 mmol) at 0 ^oC. The reaction mixture was allowed to stir at room temperature for 1 hour. After the complete conversion detected LC-MS analysis, the resulting mixture was filtered and the filtered liquid was concentrated under vacuum to remove the MeOH. Then the residual solution was neutrilized by the addtion of saturated $NaHCO_3$ aqueous solution to PH 7~8 and extracted with EA (100.0 mL * 3). The combined orgainc phases were washed with brine, dried over anhydrous Na_2SO_4, filtered, concentrated under vacuum, absorbed onto aluminum oxide, and purified via flash column chromatography (EA : hexanes = 1 : 99 to 1 : 1) to afford a black solid as 5-bromo-N^1-methylbenzene-1,2-diamine (**8**, 1.8 g, 71% yield). LC-MS: 201.01 $[M]^+$.

*Step g: synthesis of 6-bromo-1-methyl-1,3-dihydro-2H-benzo[d]imidazol-2-one, **9***

To a solution of 5-bromo-N^1-methylbenzene-1,2-diamine (**8**, 1.0 g, 5.0 mmol) in acetonitrile (20.0 mL) was added carbonyldiimidazole (4.0 g, 24.9 mmol). The reaction mixture was refluxed at 85 ^oC overnight. After cooling, the resulting mixture was concentrated under vacuum to afford a dark brown residue. The residue was partitioned between water (50.0 mL) and EA (50.0 mL * 3). The combined organic layers were washed with brine, dried over anhydrous Na_2SO_4, filtered, concentrated under vacuum, absorbed

onto silica gel, and purified via flash column chromatography (DCM : MeOH = 99 : 1 to 95 : 5) to afford a brown-orange solid as 6-bromo-1-methyl-1,3-dihydro-2*H*-benzo[*d*]imidazol-2-one (**9**, 0.5 g, 41% yield). LC-MS: 227.01 $[M]^+$.

Step h: synthesis of methyl 3-(3-methyl-2-oxo-2,3-dihydro-1H-benzo[d]imidazol-5-yl)benzoate, ***10***

A mixture of 6-bromo-1-methyl-1,3-dihydro-2*H*-benzo[*d*]imidazol-2-one (**9**, 70.0 mg, 0.3 mmol), 3-methoxycarbonylphenylboronic acid (80.1 mg, 0.45 mmol), LiCl (38.2 mg, 0.9 mmol), freshly prepared 2.5 M Na_2CO_3 aqueous solution (0.30 mL, 0.75 mmol), and bis(triphenylphosphine)palladium(II) dichloride (10.6 mg, 0.16 mmol) in toluene (4.0 mL) and EtOH (4.0 mL) was degassed with N_2 for 15 minutes. The reaction mixture was sealed in a 20.0 mL vial and stirred at 95 ^oC overnight. After the complete conversion detected LC-MS analysis, the resulting mixture was concentrated under vacuum, absorbed onto silica gel, and purified via flash column chromatography (DCM : MeOH = 99 : 1 to 10 : 1) to afford a white solid as methyl 3-(3-methyl-2-oxo-2,3-dihydro-1*H*-benzo[*d*]imidazol-5-yl)benzoate (**10**, 40.0 mg, 47% yield). LC-MS: 283.15 $[M]^+$.

Step i: synthesis of 3-(3-methyl-2-oxo-2,3-dihydro-1H-benzo[d]imidazol-5-yl)benzoic acid, ***11***

To a solution of methyl 3-(3-methyl-2-oxo-2,3-dihydro-1*H*-benzo[*d*]imidazol-5-yl)benzoate (**10**, 40.0 mg, 0.14 mmol) in MeOH (2.1 mL) and tetrahydrofuran (2.1 mL) was added a solution of $LiOH \cdot H_2O$ (8.9 mg, 0.21 mol) in water (0.7 mL). The reaction mixture was stirred at room temperature overnight. After the complete conversion detected LC-MS analysis, the resulting mixture was concentrated under vacuum to remove the organic solverts and diluted with water (2.0 mL). The aqueous solution was acidified via the addition of 2 M HCl solution in water to PH 4 to obtain a white suspension. After filtration, the off-white solid collected was washed with Et_2O (0.5 mL) to afford a white solid as 3-(3-methyl-2-oxo-2,3-dihydro-1*H*-benzo[*d*]imidazol-5-yl)benzoic acid (**11**, 30.0 mg, 80% yield). LC-MS: 269.39 $[M]^+$.

*Step j: synthesis of 3-(3-methyl-2-oxo-2,3-dihydro-1H-benzo[d]imidazol-5-yl)-N-(5,6,7,8-tetrahydroimidazo[1,2-a]pyridin-7-yl)benzamide, (**Z36-MP5**)*

A mixture of 3-(3-methyl-2-oxo-2,3-dihydro-1*H*-benzo[*d*]imidazol-5-yl)benzoic acid (**11**, 30.0 mg, 0.11 mmol), 5,6,7,8-tetrahydroimidazo[1,2-a]pyridin-7-amine dihydrochloride (**5**, 19.4 mg, 0.11 mmol), and *N*,*N*-diisopropylethylamine (0.12 mL, 0.66 mmol) in anhydrous DMF was stirred at 0 ^oC for 5 minutes. The HATU (50.2 mg, 0.13 mmol) was added in one portion. The reaction mixture was stirred at room temperature overnight. After the complete conversion detected LC-MS analysis, the resulting mixture was diluted with water (20.0 mL) and extracted with EA (20.0 mL * 3). The combined orgainc layers were washed with brine, dried over anhydrous Na_2SO_4, filtered, concentrated under vacuum, absorbed onto silica gel, and purified via flash column chromatography (DCM : MeOH = 99 : 1 to 10 : 1) to afford a white solid as 3-(3-methyl-2-oxo-2,3-dihydro-1*H*-benzo[*d*]imidazol-5-yl)-*N*-(5,6,7,8-tetrahydroimidazo[1,2-*a*]pyridin-7-yl)benzamide (**12**, 10.2 mg, 23.9% yield). LC-MS: 388.09 $[M+H]^+$. Source data are provided as a Source Data file.
