# Supplementary material for: Mi-2β promotes immune evasion in melanoma by activating EZH2 methylation
Source: Nat Commun. 2024 Mar 9;15:2163. doi: 10.1038/s41467-024-46422-5 (PMC10924921; doi:10.1038/s41467-024-46422-5)
Supplement: Supplementary file 3 — Description of Additional Supplementary Files [file 41467_2024_46422_MOESM3_ESM.pdf]

Description of Additional Supplementary Files

File Name: Supplementary Data 1
Description: Identification of CHD4 interacting proteins

File Name: Supplementary Data 2
Description: Profile of Z36-MP5 inhibition on ATPases

File Name: Supplementary Data 3
Description: gRNA and qRCR primer sequences and antibody information
